# Hybrid Imaging in Head and Neck Sarcoidosis

**DOI:** 10.3390/jcm8060803

**Published:** 2019-06-05

**Authors:** Isidora Grozdic Milojevic, Marijana Tadic, Dragana Sobic-Saranovic, Jelena Saponjski, Vera M. Artiko

**Affiliations:** 1 Center for Nuclear Medicine, Clinical Center of Serbia, 11000 Belgrade, Serbia; drisidora.grozdic@yahoo.com (I.G.M.); d.sobic@gmail.com (D.S.-S.); jelena.saponjski@med.bg.ac.rs (J.S.); artiko@med.bg.ac.rs (V.M.A.); 2 Faculty of Medicine, University of Belgrade, 11000 Belgrade, Serbia; 3 Department of Internal Medicine and Cardiology, Charité-University-Medicine Berlin, Campus Virchow Klinikum (CVK), 13353 Berlin, Germany

**Keywords:** FDG PET/CT, chronic sarcoidosis, head and neck sarcoidosis

## Abstract

To determine the prevalence of head and neck sarcoidosis (HNS) and evaluate the role of hybrid molecular imaging in HNS. Between 2010 and 2018, 222 patients with chronic sarcoidosis and presence of prolonged symptoms of active disease were referred to FDG PET/CT. Active disease was found in 169 patients, and they were all screened for the presence of HNS. All patients underwent MDCT and assessment of the serum ACE level. Follow-up FDG PET/CT examination was done 19.84 ± 8.98 months after the baseline. HNS was present in 38 out of 169 patients. FDG uptake was present in: cervical lymph nodes (38/38), submandibular glands (2/38), cerebrum (2/38), and bone (1/38). The majority of patients had more than two locations of disease. After FDG PET/CT examination, therapy was changed in most patients. Fourteen patients returned to follow-up FDG PET/CT examination in order to assess the therapy response. PET/CT revealed active disease in 12 patients and complete remission in two patients. Follow-up ACE levels had no correlation with follow-up SUVmax level (*ρ* = −0.18, *p* = 0.77). FDG PET/CT can be useful in the detection of HNS and in the evaluation of the therapy response. It may replace the use of non-purposive mounds of insufficiently informative laboratory and radiological procedures.

## 1. Introduction

Sarcoidosis is a systemic granulomatous disease of unknown cause. The micro-architecture of the granuloma results from CD4 T cell mediated activation of macrophages, which undergo epithelioid transformation and can also form multinucleated giant cells. The restriction of the T cell receptor repertoire supports the hypothesis that sarcoidosis is an antigen driven process even though the antigen remains unknown [1,2].

Numerous studies suggest that disease can occur after exposure to pollen or exposure to *Mycobacterium tuberculosis*. Other studies assume that sarcoidosis causes exposure to some chemicals and insecticides (occupational disease). On the other hand, some authors believe that disease is genetically predisposed and more frequent in the HLA-B8/A1 gene locus population [1].

Regardless of the cause of disease, sarcoidosis has been reported in every system and organ of the human body. Thoracic localization is most commonly involved, while extra-thoracic is present in 25% to 50% of the cases. An extratoracic disease can give an atypical symptomatology, which can be associated with more frequent diseases. This frequency results in a diagnosis that is delayed by months to years [2,3,4].

Head and neck sarcoidosis may occur in combination with or independently of CNS sarcoidosis and has been shown to be present in 10% to 15% of patients with systemic disease [5]. Any structure within the head and neck can be involved, such as: skin, neck, larynx, salivary glands, cervical lymph nodes, oropharynx, hypopharynx, sinuses, eye, bone, and even intracranial infiltration. Once a patient begins having head and neck involvement, the otolaryngologist is often involved both for diagnostic and interventional purposes [6].

Occasionally, rare misleading manifestations, although within the realm of sarcoidosis manifestations, can lead to sarcoidosis being missed among the gamut of possible diagnoses by physicians inexperienced in recognizing sarcoidosis, particularly in elder patients [3].

Until today, the exact prevalence of head and neck sarcoidosis is unknown due to different results depending on the studied population, used diagnostic techniques, and many asymptomatic patients. Manifestations within the head and neck along with other extrathoracic manifestations are associated with more extensive disease requiring coordinated treatment. Therefore, it is important to understand rare manifestations of sarcoidosis as they can quickly involve vital structures [6,7]. Awareness of the imaging findings of head and neck sarcoidosis would help prevent long-standing unrecognized disease.

One should not forget the fact that this disease mostly affects the working population, which is why it is necessary to assess the presence of active disease in an adequate manner. This method would enable timely treatment of the disease and reduce the disability and costs of treating the complications of the disease.

The gold standard for diagnosis of sarcoidosis is still patho-histology [2,8]. Assessment of activity and the extent of disease remain a clinical challenge, since the most common laboratory parameters (serum level of angiotensin converting enzyme—ACE) have low sensitivity and specificity [9]. On the other hand, standard radiology techniques such as multi-detector computer tomography (MDCT) and magnetic resonance cannot reveal active inflammation [2,10].

Currently, inflammatory activity can be evaluated routinely with hybrid imaging (positron emission tomography with computed tomography using 18F-fluorodeoxyglucose—FDG PET/CT), which allows the information of precise anatomic localization of the disease and the functional changes at the cellular level.

High FDG uptake corresponds to increased glucose uptake and consumption through the hexose monophosphate shunt, which is the main source of energy for chemotaxis and phagocytosis. ^18^F-FDG, which is a glucose analog, is taken up by living cells via the cell membrane glucose transporters and is, subsequently, phosphorylated with hexokinase. Activation of phagocytes (“respiratory burst activation”) brings increased ^18^F-FDG uptake. Increased ^18^F-FDG uptake is present in neutrophils during the acute phase of inflammation, whereas macrophages and polymorphonuclear leukocytes uptake ^18^F-FDG is present during the chronic phase. Thus, inflammation and infection can be evaluated in this way [11,12,13].

However, the role of FDG PET/CT in head and neck sarcoidosis has not been evaluated enough, but it is reported in case reports and descriptive articles [3,6,14,15,16,17,18,19,20]. To the best of our knowledge, until now, no prospective study has been done in this field. Therefore, the aim of this study was to determine the prevalence of head and neck sarcoidosis and evaluate the role of hybrid molecular imaging in these patients.

## 2. Materials and Methods

### 2.1. Study Population

This prospective study included 330 consecutive patients with chronic sarcoidosis referred for FDG PET/CT examination at the National PET Center at the Clinical Center of Serbia between January 2010 and December 2018.

Inclusion criteria for this study were: chronic sarcoidosis confirmed by biopsy and the presence of prolonged symptoms or new clinical, biochemical, or imaging findings suggestive of active disease. Exclusion criteria were: presence of cancer or other diseases that resemble sarcoidosis on MDCT and PET/CT (Wegener syndrome, tuberculosis, aspergillosis), existence of head and neck infection, and the glucose level being greater than 11 mmol/L [19]. Out of 330 referred patients, seven were excluded because of the detected cancer (two breast cancer, two Hodgkin lymphoma, one lung cancer, one cervical cancer, and one rectal carcinoma), and one patient had tuberculosis in the patient’s medical history. Thus, the final sample consisted of 222 patients (mean age 48.88 ± 12.10 years, 85 men and 137 women). Then, FDG PET/CT positive patients were screened for the presence of abdominal sarcoidosis. The Ethics Committee of the Faculty of Medicine of the University of Belgrade approved the study protocol, and the written informed consent was obtained.

### 2.2. Procedures

Before FDG PET/CT examination, all subjects underwent standard radiography and high-resolution CT of the chest and serum measurement of the ACE level (reference range, 8–52 U/L). Based on the baseline FDG PET/CT findings, 38 out of 222 patients had positive findings for head and neck sarcoidosis. After the first FDG PET/CT examination, physicians changed the therapy in all patients, and they were invited to participate in the follow-up FDG PET/CT study. Fourteen patients returned for the follow-up FDG PET/CT examination, which was performed 19.84 ± 8.98 months after the baseline. The follow-up ACE data were extracted from the medical records.

### 2.3. Data Acquisition, Reconstruction, and Image Analysis

FDG PET/CT examination was performed on a 64-slice hybrid PET/CT scanner (Biograph, Siemens Medical Solutions USA Inc.). Patients fasted for 8 h before receiving an intravenous injection of 5.50 MBq of 18F-FDG per kilogram. PET/CT acquisitions started 60 min after tracer injection. A 3-dimensional PET scan (6–7 fields of view-, 3 min/field, 30% overlap of beds, the bed width of ~16 cm) and low-dose nonenhanced CT scan were acquired from the skull to the mid-thigh. If deemed necessary, a total-body study was also performed. The PET/CT scanner was not dynamic, the trans-axial field of view was 605 mm, and the axial field of view was 162 mm. The reconstruction method was iterative with 4 iterations and 8 subsets, the matrix 168 × 168, the zoom 1.0. The Gaussian filter was used with FWHM (Full Width at Half Maximum) 5 mm. MDCT was acquired with 120 kV and with automatic, real-time dose modulation amperage (CareDose4D (Siemens, Munich, Germany), with the baseline being 45 mA), the slice thickness of 5 mm, the pitch of 1.50, and the rotation time of 0.50 s. Low-dose CT, attenuation corrected PET, and combined PET/CT images were displayed for analysis on a syngo Multimodality Workplace (Siemens AG). Currently FDG PET/CT is used as a tool for the detection of active sites of sarcoidosis, which relies on qualitative assessment with visual interpretation and quantitative analysis. Semi-quantitatively expressed, this leads to dichotomous “positive” or “negative” results. “Positive” results were above the background level (blood pool and surrounding normal lung parenchyma and/or lymph node activity above the blood pool activity) [16,21]. Quantitative analysis of FDG uptake in the lesion was done based on the maximum standardized uptake value per focus (SUVmax). This value was calculated as the activity concentration measured at the end of the scan and corrected for individual body weight and dose injected as follows: tissue activity (counts/pixel/s) multiplied by the calibration factor and divided by an injected FDG dose (MBq/kilogram of body weight) [22]. In our study (as well as in most other studies that evaluate sarcoidosis activity) SUVs were calculated (as in oncology) using the maximum voxel (SUVmax) within the ROI drawn around each lesion using 42% or more of the maximum voxel as the sampled volume [16,21].

The follow-up FDG PET/CT was done identically in terms of the dose given, wait time, data acquisition, reconstruction, and image analysis. FDG PET/CT data were interpreted separately by three investigators including two nuclear medicine physicians and a radiologist. Consensus was reached in cases of discrepancy.

### 2.4. Statistical Analysis

Pearson’s correlation test was used in order to assess correlation between SUVmax levels and ACE levels on the baseline and follow-up examination. For testing the difference between dichotomous variables on the follow-up examination (therapy, PET/CT findings (positive or negative)), the Fischer exact test/*X*^2^ test was used. We used the paired t-test to compare SUVmax levels between the baseline and follow-up in 14 subjects who underwent two FDG PET/CT evaluations. Changes in the ACE levels were also compared with the paired *t*-test. The results were presented as mean ± standard deviation (SD) and *p*-values of less than 0.05 was considered significant.

## 3. Results

### 3.1. Patient Characteristics and Baseline FDG PET/CT Findings

We evaluated 222 patients with chronic sarcoidosis referred for FDG PET/CT examination (mean age 48.88 ± 12.10 years, 85 men and 137 women). The clinical diagnosis of sarcoidosis was given prior to PET/CT examination. It was done after the evaluation of medical examination, laboratory analyses (including ACE, chitotyrosidase), and supplemental analysis (CT, MR (Magnetic resonance), pulmonary spirometry, heart ultrasound, ECG (electrocardiogram)). Depending on the symptoms of disease, consultations were requested from other specialists. The final diagnosis was made after pathohistological verification. The samples were taken from susceptible sites (depending on the location of abnormalities, the biopsy was taken from skin/lymph nodes/abdomen/liver etc. or during bronchoscopy). All patients referred to FDG PET/CT examination were screened first for positive findings. Active disease was found in 169 (76%) patients. Head and neck sarcoidosis was present in 38 (17%) patients. Eighteen men and 20 women (mean age 49.11 ± 12.13 years) had active disease in the head and neck region. The majority of them also had active disease in the thorax (96%), whereas isolated extrathoracic localization was less frequent (4%). Most of the patients had nonspecific symptoms of disease (fatigue, fever, headache, etc.) and high ACE level (44.28 ± 12.55 U/L) (see Table 1).

**Figure 1:**
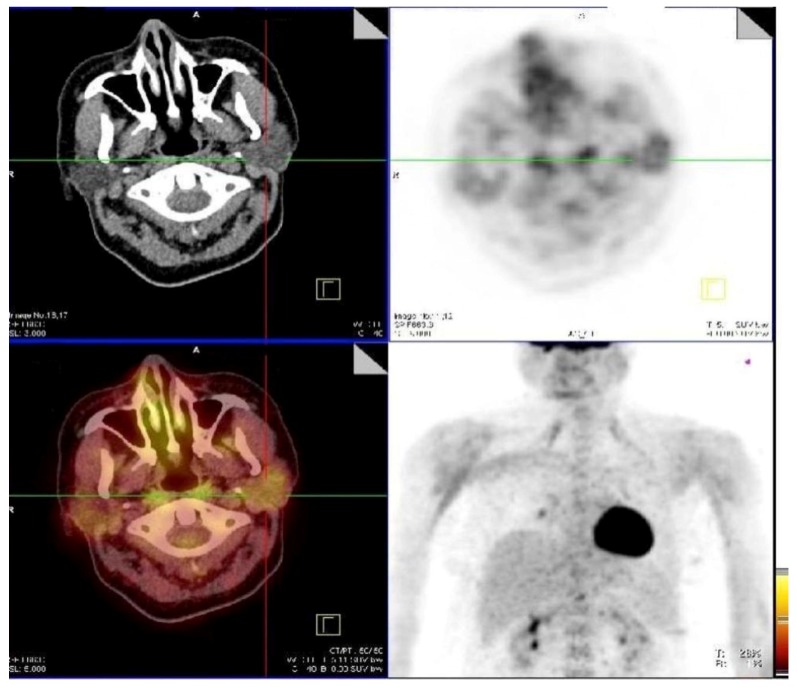
Active sarcoidosis present in submandibular glands, right supraclavicular lymph node, right hylar lymph node.

**Figure 2:**
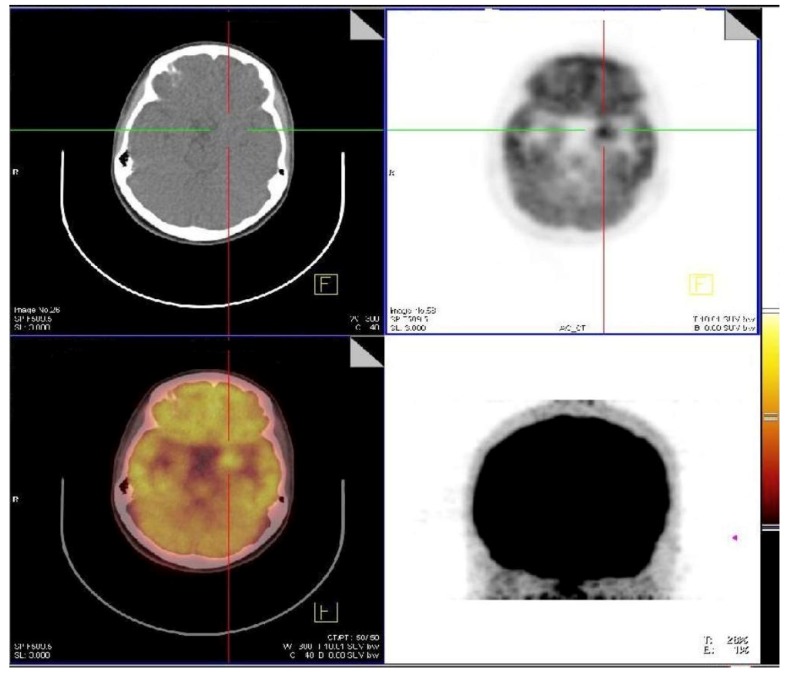
Active neurosarcoidosis present in left temporo-mesial region.

**Figure 3:**
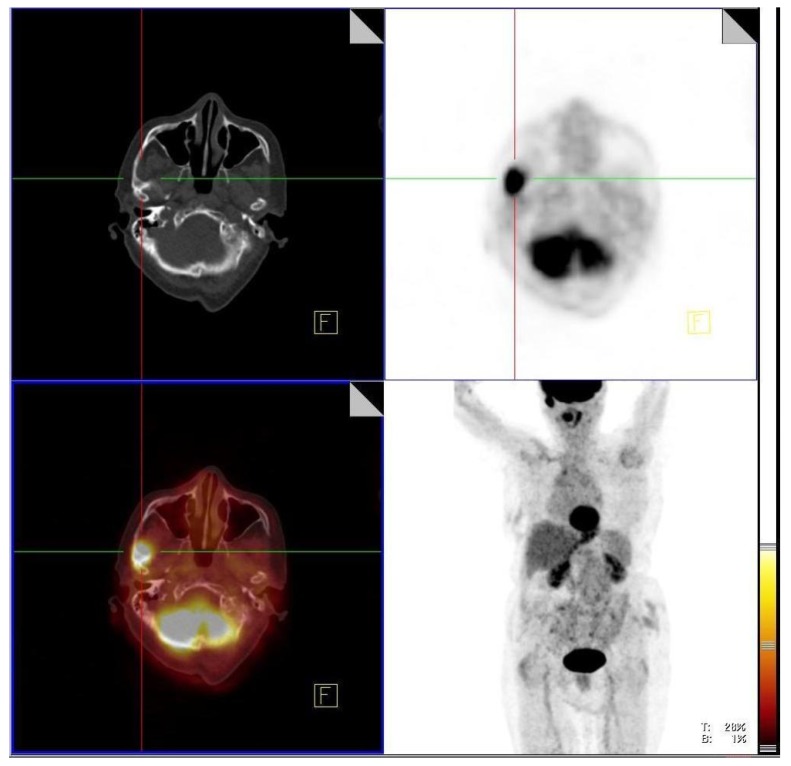
Active sarcoidosis present in right zygomatic bone.

The most frequent localizations of head and neck sarcoidosis were cervical lymph nodes, which were present in all patients, whereas less frequent localization were submandibular glands (2/38) in Figure 1, cerebrum (2/38) in Figure 2, and bone (1/38) in Figure 3. Although most of the patients had elevated ACE levels, they were not in correlation with the SUVmax level (*ρ* = −0.04, *p* = 0.83).

### 3.2. Changing of Therapy and Follow-Up FDG PET/CT Findings

After the first FDG PET/CT examination, the referring physicians changed the therapy in the majority of patients with head and neck sarcoidosis based on clinical, biochemical, and imaging indicators of disease activity. Higher corticosteroid doses were given to eight patients. Two patients were given methotrexate along with pronisone, whereas, in three patients, the therapy was not changed (since they had localized and moderately active disease).

**Figure 4:**
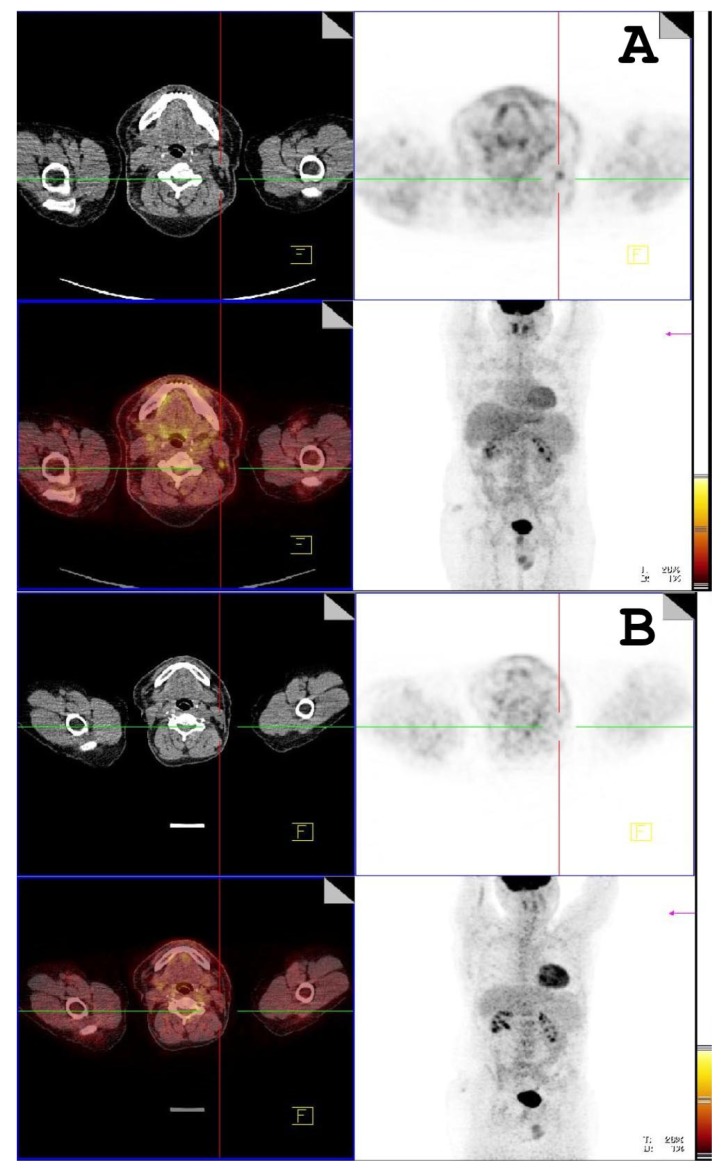
(**A**) First FDG PET/CT scan, active disease (sarcoidosis) present in the left cervical lymph node (**B**) Follow-up FDG PET/CT scan, total remission of disease after therapy.

After 19.84 ± 8.98 months, 14 patients (37%) returned to follow-up FDG PET/CT examination in order to assess the therapy response. Follow-up PET/CT revealed active disease in 12 patients. Follow-up SUVmax was similar to the first one in the majority of the patients (10.14 ± 5.44 vs. 10.53 ± 5.04, *p* = 0.816). Complete remission of disease was present in only two patients (see Figure 4). On follow-up FDG PET/CT examination, ACE levels decreased (44.28 ± 12.55 vs. 47.02 ± 19.50, *p* = 0.80), but the reduction was not statistically significant. Follow-up ACE levels had no correlation with the follow-up SUVmax level (*ρ* = −0.18, *p* = 0.77).

## 4. Discussion

This study prospectively examined the prevalence of head and neck sarcoidosis and the usefulness of FDG PET/CT examination. FDG PET/CT detected head and neck sarcoidosis in 17% of the patients with active disease. Our results also suggest that FDG PET/CT can be used in evaluation of the therapy response in patients with head and neck sarcoidosis, which should provide the best possible treatment in these patients. Prabahar et al. cited similar findings [7].

The role of FDG PET/CT in the detection of head and neck sarcoidosis is poorly described in previous case reports and studies [3,6,14,15,17,18,19]. To the best of our knowledge, no prospective study has been done in this field. The prevalence of head and neck sarcoidosis in our study (17%) agrees with the rate previously reported [3]. As well as the previous reports, our results showed that cervical lymph nodes were the most frequent localization of head and neck sarcoidosis (present in all cases in our study population). Lymphadenopathy was usually present in the posterior triangle. Calcification of lymph nodes was sometimes seen, but less than in mediastinum. During the evaluation of increased FDG accumulation in the lymph nodes, one should always keep in mind that other conditions can give a similar presentation of disease (reactive lymph nodes, lymphoma, nodal metastases, or other granulomatous diseases) [3,23,24,25].

Next, frequent location of head and neck sarcoidosis were salivary glands. Their involvement was seen in 5% of our study population. The results are concordant with Morowka-Kata and Vourexakis studies and results [26,27]. Parotid gland swelling was the most common manifestation of disease. Unilateral or bilateral parotid gland enlargement could be in the setting of viral or bacterial process (acute /chronic sialodenitis) and should be excluded, since it may give the same presentation in the FDG PET/CT imaging.

Bone involvement is a rare manifestation of disease in the head and neck. In our study population, it was seen in only one patient with focal increased FDG uptake in the zygomatic bone.

Neurosarcoidosis was also rare, which is present in only two patients from our study population. The previous descriptive study of Ianuzzi et al. showed a slightly higher percentage of neurosarcoidosis [1]. The difference between the results can be explained by the differences in diagnostic procedures (FDG PET/CT vs. MR). The gadolinium-enhanced MR is a modality of choice for evaluation of neurologic involvement because of its superior soft-tissue discrimination. On the other hand, normal uptake of FDG is seen in cerebral parenhima, which can aggravate the interpretation of PET/CT in this region. The most typical presentation of neurosarcoidosis is a headache and encephalopathy with seizures due to meningeal involvement. Cranial nerve palsy is also a commonly presented feature. Extension of leptomeningeal sarcoid into the perivascular spaces results in ischemic or inflammatory damage to the surrounding brain. Granulomatous inflammation causes hypermetabolism, and neuronal damage produces hypometabolism [28,29,30]. Diagnosing sarcoidosis in the head and neck is challenging because of its varied clinical manifestations and imaging appearances that are similar to those of other pathiologic entities. Neurosarcoidosis requires confirmatory tissue biopsy. In some patients, biopsy can be difficult, such as in those with neurological disease and those with involvement at sites with limited accessibility.

Sarcoidosis is a disease that often requires long-term treatment. Unfortunately, the therapeutic modality in the treatment of sarcoidosis is limited to the use of corticosteroids with the possible addition of antimetabolite (methotrexate, azathioprine) or TN F-α antagonists. Long-term use of corticosteroids carries a number of adverse effects of this therapy [1].

It is of particular importance to find an adequate diagnostic procedure to monitor the activity and the extent of disease in order to have a more accurate picture of the patient’s condition on the basis of which the first-time therapy would be planned. It is also extremely important to monitor the effect of the therapy. 

Hybrid imaging procedures (PET/CT, PET/MR) take precedence over radiological ones (MDCT, MR) because they notice functional changes at the molecular level long before anatomical changes occur. Additionally, semi-quantification of accumulation of radioactive contrast is objectively assessing the therapeutic response. Thus, patients would receive a therapy that would correspond to their subjective condition [31,32,33,34].

**Figure 5:**
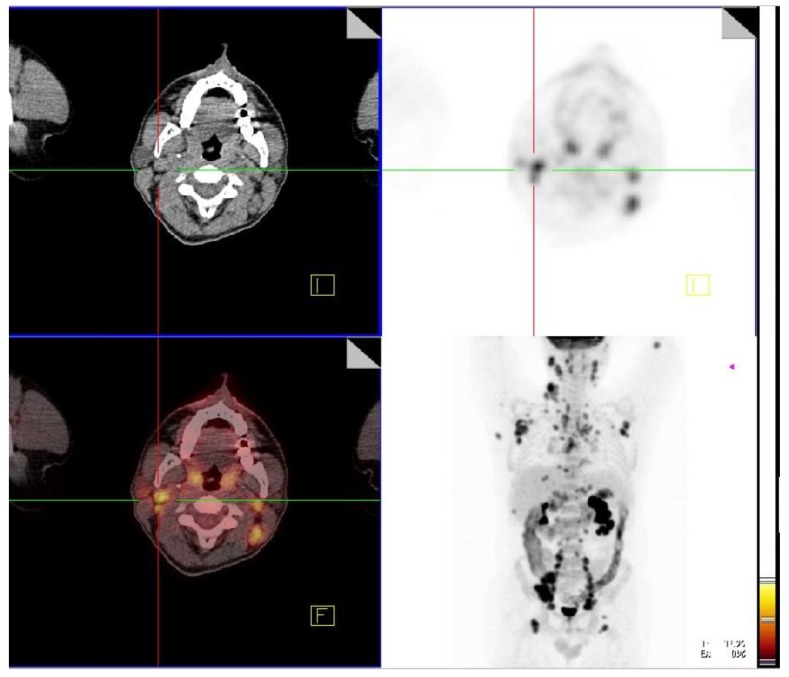
Wide spread disease, active sarcoidosis in cervical lymph nodes, axilar lymph nodes, mediastinal lymph nodes, lungs, liver, spleen, retroperitoneal and paraortal lymph nodes.

In this study, FDG PET/CT revealed the presence of active and widespread disease in the majority of the patients (Figure 5), after which the therapy was changed in most patients. Follow-up FDG PET/CT showed a variable response to treatment across the group. Follow-up SUVmax levels were similar to the initial ones in the majority of the patients. Only two patients had complete remission of disease during the follow-up, whereas others mostly had disease that was less spread out. Likely, a more significant decrease in SUVmax levels will be expected in a longer follow-up period, during which the effects of the therapy would be fully manifested [35].

Laboratory tests are not of key importance in the evaluation of disease activity. Our study results re-affirm low sensitivity of ACE, which was not consistent with the symptoms of the disease nor with the activity and the extent of the disease [22,31,32,33,34,35].

The advantage of FDG PET/CT, hybrid imaging, is providing information of precise anatomic localization of the disease and the functional changes at the cellular level. Additionally, the whole-body study protocol is especially important in order to evaluate the extent of this multisystem disease [7]. 

Hybrid imaging is more expensive than CT scan. Nevertheless, the higher expense is well balanced by its ability to demonstrate activity/inactivity of the phlogistic foci, which is not possible with morphologic examinations. However, we have to take into account that the radiation burden of a high resolution or a contrast-enhanced CT scan is accompanied by a greater radiation burden than the one necessary in the PET/CT scan [36]. When using a low dose, CT protocol optimization resulted in a 32% reduction of the mean CT radiation dose. The mean effective dose was reduced from 8.10 to 5.50 mSv. The blinded analysis of the image quality showed no clinically significant degradation of the lower-dose studies [36].

Hybrid imaging may replace the use of non-purposive mounds of laboratory and radiological procedures, which are not sufficiently informative in assessing the activity of inflammatory disease in patients with sarcoidosis [12].

This study has several limitations. A potential limitation is a relatively small follow-up sample size (14/38 pts). Patients who did not come to the follow-up examination mostly lived in distant cities, and they were treated in local hospitals. However, to our best knowledge, this is the largest follow-up sample size with abdominal sarcoidosis evaluated with FDG PET/CT. Furthermore, an observation period (19.84 ± 8.98 months) may be potentially short to fully evaluate the effects of corticosteroid therapy [35]. Future studies should be done in a larger sample, with a longer follow-up.

Additionally, the use of quantification using the SUVmax has its drawbacks. Even in the same patient on two consecutive days, it can vary by 10% to 20%, because the amount of FDG uptake in a lesion is dependent on multiple biologic and technical factors (blood glucose levels, time between FDG injection and image acquisition, etc.) [21]. Currently, various automated methods are used to quantitatively estimate FDG uptake: fixed SUV threshold (e.g., SUV2.50), percentage threshold of SUVmax, gradient-based threshold, and background-related threshold [37]. New volumetric quantification methods such as: percentage of lung volume with increased metabolic activity (“%SUV-high”) and average metabolic activity in the lung (“SUVmean”) could be helpful for evaluating small lung changes. However, new studies should confirm their usefulness [38].

Another limitation is that not all patients had histological proof of head and neck sarcoidosis. Before FDG PET/CT examination, all patients had pathohistological verification of disease, with biopsies in other easily available regions. Since we excluded patients with malignancy, other granulomatous and inflammatory diseases that could have a similar appearance on FDG PET/CT as sarcoidosis, intensive FDG uptake in the head and neck was interpreted as the presence of active disease. The decision was made more easily based on the fact that most patients had an active disease in the thorax.

## 5. Conclusions

Hybrid imaging (FDG PET/CT) can be a useful tool for detecting head and neck sarcoidosis. In this study population, the prevalence of disease was 17%, and cervical lymph nodes were the most frequently affected site (100%), which is followed by the salivary gland (5%). Although the cost of a PET/CT scan is currently high, the test provides whole-body dynamic physiological information unattainable with other radiological procedures [12]. This can be helpful for head and neck sarcoidosis in deciding the biopsy sites and monitoring the therapeutic response. Awareness of the imaging findings of head and neck sarcoidosis would help prevent long-standing, unrecognized disease. This approach holds potential in evaluating disease severity, by replacing the more traditional but less sensitive ACE test. Benefits of hybrid imaging over conventional PET are evident when taking into account the usefulness of the obtained precise anatomical localization of the changes. Using a low dose CT protocol, significant optimization of the radiation dose can be obtained. Further studies using PET/MR may further decrease the radiation dose, and bring additional information about the pathology of the central nervous system. New prospective multicenter studies are necessary to confirm these findings and define the clinical role of FDG PET/CT in head and neck sarcoidosis.

## Figures and Tables

**Table 1:** Characteristics of study population.

	Study Population (*n* = 38)
Mean age (year)	49.11 ± 12.13
Gender (Female)	20 (52.6%)
ACE (mean ± SD, U/L)	44.28 ± 12.55
SUVmax (mean ± SD)	10.53 ± 5.04
Therapy (yes) Prednisone Prednisone and methotrexate	13 (48%) 12 (44%) 1 (4%)

## List of Abbreviations

ACE	Angiotensin converting enzyme
MDCT	Multi detector computer tomography
FDG PET/CT	Positron emission tomography with computed tomography using 18F-fluorodeoxyglucose
SUVmax	Maximum standardized uptake value per focus
SD	Standard deviation
